# *N*,*N*-Bis(diphenyl­phosphino)-1,2-dimethyl­propyl­amine

**DOI:** 10.1107/S1600536808001839

**Published:** 2008-01-23

**Authors:** Nicoline Cloete, Hendrik G. Visser, Andreas Roodt, Jontho T. Dixon, Kevin Blann

**Affiliations:** a Department of Chemistry, University of the Free State, PO Box 339, Bloemfontein 9300, South Africa; b R&D Division, Sasol Technology (Pty) Ltd, 1 Klasie Havenga Road, Sasolburg 1947, South Africa

## Abstract

The diphenyl­phosphine groups in the title compound, C_29_H_31_NP_2_, are staggered relative to the PNP backbone. The N atom adopts an almost planar geometry with the two P atoms and the C atom attached to it, in order to accommodate the steric bulk of the phenyl groups and the alkyl group. Three C atoms of the 1,2-dimethylpropylamine group are disordered over two positions in a 9:1 ratio. The mol­ecules pack diagonally in the unit cell across the *ac* plane in a head-to-tail fashion.

## Related literature

For similar structures, see: Keat *et al.* (1981 ▶); Cotton *et al.* (1996 ▶); Fei *et al.* (2003 ▶). For ethyl­ene tetra­merization, see: Bollmann *et al.* (2004 ▶).
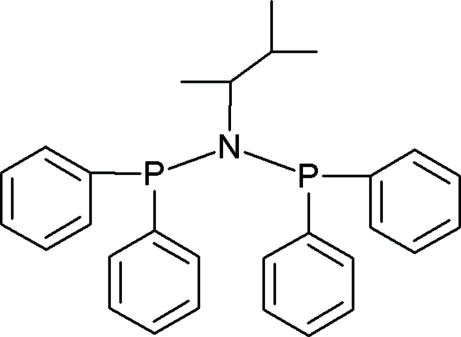


## Experimental

### 

#### Crystal data

C_29_H_31_NP_2_
*M* *_r_* = 455.49
Triclinic, 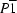

*a* = 9.242 (5) Å
*b* = 10.454 (5) Å
*c* = 12.899 (5) Å
α = 91.031 (5)°
β = 98.188 (5)°
γ = 102.775 (5)°
*V* = 1201.4 (10) Å^3^
*Z* = 2
Mo *K*α radiation
μ = 0.20 mm^−1^
*T* = 101 (2) K
0.47 × 0.29 × 0.14 mm

#### Data collection

Bruker Kappa APEXII diffractometer
Absorption correction: multi-scan (*SADABS*; Bruker, 2004 ▶) *T*_min_ = 0.912, *T*_max_ = 0.974
24124 measured reflections
5947 independent reflections
5313 reflections with *I* > 2σ(*I*)
*R*_int_ = 0.031

#### Refinement

*R*[*F* ^2^ > 2σ(*F* ^2^)] = 0.040
*wR*(*F* ^2^) = 0.107
*S* = 1.09
5947 reflections
299 parameters
H-atom parameters constrained
Δρ_max_ = 0.56 e Å^−3^
Δρ_min_ = −0.52 e Å^−3^

### 

Data collection: *APEX2* (Bruker, 2005 ▶); cell refinement: *SAINT-Plus* (Bruker, 2004 ▶); data reduction: *SAINT-Plus*; program(s) used to solve structure: *SIR97* (Altomare *et al.*, 1999 ▶); program(s) used to refine structure: *SHELXL97* (Sheldrick, 2008 ▶); molecular graphics: *DIAMOND* (Brandenburg & Putz, 2005 ▶); software used to prepare material for publication: *WinGX* (Farrugia, 1999 ▶).

## Supplementary Material

Crystal structure: contains datablocks global, I. DOI: 10.1107/S1600536808001839/pv2057sup1.cif

Structure factors: contains datablocks I. DOI: 10.1107/S1600536808001839/pv2057Isup2.hkl

Additional supplementary materials:  crystallographic information; 3D view; checkCIF report

## Figures and Tables

**Fig. 1.**
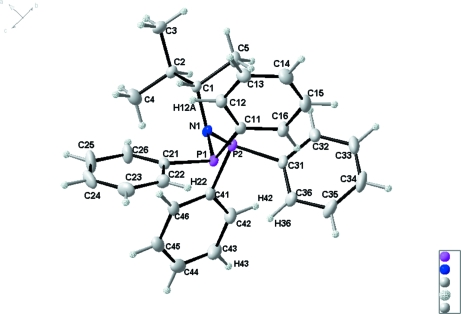
A view of (I) shown with 50% probability displacement ellipsoids. Atoms C1', C2' and C3' and the H atoms have been omitted for clarity.

**Fig. 2.**
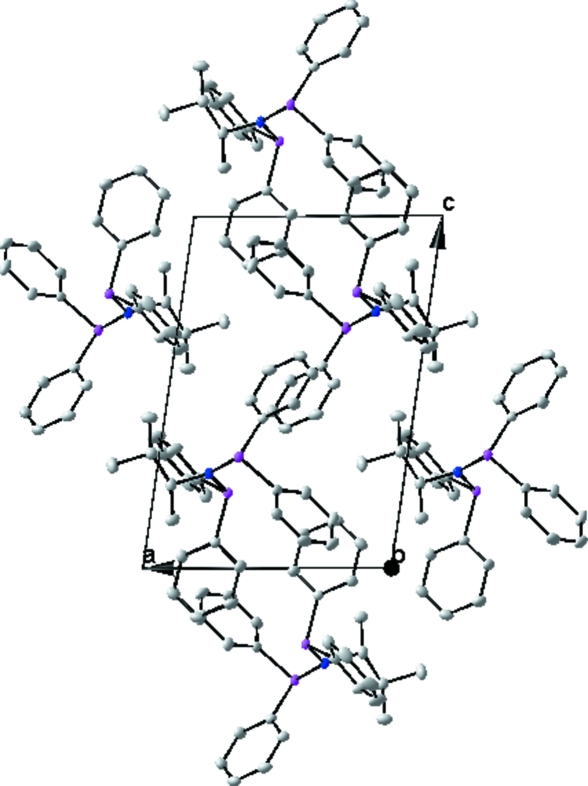
A view of (I) illustrating the disordered part of the molecule.

**Fig. 3.**
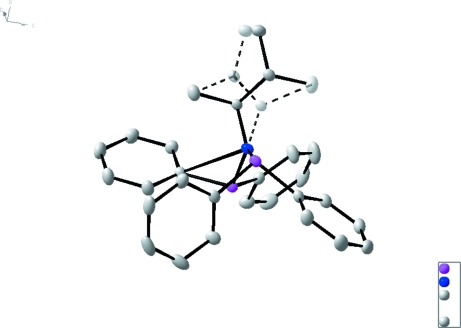
A perspective view of the unit cell of (I) along the b axis.

## supplementary crystallographic information

### Comment

Diphosphinoamine (PNP) ligands with various substituents on both the P and N atoms have proven to be very effective in ethylene tetramerization catalyst systems and have been shown to produce 1-octene in good selectivity (Bollmann *et al.*, 2004). It seems that the substituents on the N atom profoundly affected the catalyst productivity. This paper forms part of a structural and kinetic investigation into the mechanism of the catalytic cycle.

The crystals of the title compound, (I), crystallize in the triclinic space group. All bond distances and angles in (I) (Figure 1a) are considered to be normal and fall within the range reported for similar complexes (Keat *et al.*, 1981; Cotton *et al.*, 1996; Fei *et al.*, 2003). A slight disorder of 12% is observed in the 1,2-dimethylpropylamine substituent (Figure 1 b). The distance of N1 from the P1—P2—C1 plane was calculated as 0.216 (2) Å. The geometry around the phosphorous ligands is distorted from tetrahedral geometry with C—P—C angles being the most distorted (varying from 99.56 (7)° to 99.78 (7)°). The P1—N1—P2 angle is 117.83 (7)°.

### Experimental

1,2-Dimethylpropylamine (0.01 mole, 0.811 g) was dissolved in dichloromethane (30 ml) and placed in an ice bath. Triethylamine (0.03 mol, 4.22 ml) was added to the solution while it was being stirred. Chlorodiphenylphosphine (0.02 mol, 3.62 ml) was slowly added to the reaction mixture. The ice bath was removed after 30 minutes and the reaction mixture was allowed to stir at room temperature for a further 12 hrs.

The dichloromethane was removed under reduced pressure. A mixture of hexane (20 ml) and toluene (2 ml) was added to the remaining white powder and was passed through a column containing neutral activated alumina (35 g). The solvent of the eluent was removed under reduced pressure and the white precipitate was collected. The product was recrystallized from methanol, single colourless crystals were obtained (yield 2.551 g, 56.1%) the next day which were suitable for X-ray crystallography.

### Refinement

The methyl, methine and aromatic H atoms were placed in geometrically idealized positions at C—H = 0.98, 1.00, 0.95 Å, respectively, and constrained to ride on their parent atoms, with *U*_iso_(H) = 1.5*U*_eq_(C) for methyl and 1.2*U*_eq_(C) for the rest of the H-atoms. The 1,2-dimethylpropylamine substituent was disordered over two sites with site occupancy factors for atoms C1, C2 and C3 being 0.880 (3) and 0.120 (3) for the unprimed and primed atoms, respectively.

### Crystal data

**Table:**

C_29_H_31_NP_2_	*Z* = 2
*M**_r_* = 455.49	*F*_000_ = 484
Triclinic, *P*1	*D*_x_ = 1.259 Mg m^−^^3^
Hall symbol: -P 1	Mo *K*α radiation λ = 0.71069 Å
*a* = 9.242 (5) Å	Cell parameters from 5808 reflections
*b* = 10.454 (5) Å	θ = 2.5–28.3º
*c* = 12.899 (5) Å	µ = 0.20 mm^−^^1^
α = 91.031 (5)º	*T* = 101 (2) K
β = 98.188 (5)º	Needle, colourless
γ = 102.775 (5)º	0.47 × 0.29 × 0.14 mm
*V* = 1201.4 (10) Å^3^	

### Data collection

**Table:**

Bruker X8 APEXII 4K Kappa CCD diffractometer	*R*_int_ = 0.031
ω and φ scans	θ_max_ = 28.3º
Absorption correction: multi-scan(SADABS; Bruker, 2004)	θ_min_ = 1.6º
*T*_min_ = 0.913, *T*_max_ = 0.974	*h* = −12→12
24124 measured reflections	*k* = −13→13
5947 independent reflections	*l* = −17→17
5313 reflections with *I* > 2σ(*I*)	

### Refinement

**Table:**

Refinement on *F*^2^	H-atom parameters constrained
Least-squares matrix: full	*w* = 1/[σ^2^(*F*_o_^2^) + (0.0452*P*)^2^ + 0.8476*P*] where *P* = (*F*_o_^2^ + 2*F*_c_^2^)/3
*R*[*F*^2^ > 2σ(*F*^2^)] = 0.040	(Δ/σ)_max_ = 0.001
*wR*(*F*^2^) = 0.108	Δρ_max_ = 0.56 e Å^−^^3^
*S* = 1.09	Δρ_min_ = −0.52 e Å^−^^3^
5947 reflections	Extinction correction: none
299 parameters	

### Special details

**Table:**

Experimental. The intensity data was collected on a Bruker X8 Apex II 4 K Kappa CCD diffractometer using an exposure time of 20 s/frame. A total of 1264 frames were collected with a frame width of 0.5° covering up to θ = 28.27° with 99.7% completeness accomplished.
Geometry. All e.s.d.'s (except the e.s.d. in the dihedral angle between two l.s. planes) are estimated using the full covariance matrix. The cell e.s.d.'s are taken into account individually in the estimation of e.s.d.'s in distances, angles and torsion angles; correlations between e.s.d.'s in cell parameters are only used when they are defined by crystal symmetry. An approximate (isotropic) treatment of cell e.s.d.'s is used for estimating e.s.d.'s involving l.s. planes.

### Fractional atomic coordinates and isotropic or equivalent isotropic displacement parameters (Å^2^)

**Table:**

	*x*	*y*	*z*	*U*_iso_*/*U*_eq_	Occ. (<1)
P1	0.70056 (4)	0.12612 (3)	0.21511 (3)	0.01317 (9)	
P2	0.67790 (4)	0.37407 (4)	0.31828 (3)	0.01449 (9)	
N1	0.78800 (13)	0.27976 (12)	0.27060 (10)	0.0140 (2)	
C1	0.93360 (17)	0.35565 (16)	0.23925 (13)	0.0135 (3)	0.880 (3)
H1	0.9915	0.2902	0.2213	0.016*	0.880 (3)
C2	1.03165 (18)	0.44779 (17)	0.33011 (13)	0.0161 (3)	0.880 (3)
H2	0.9765	0.5164	0.3455	0.019*	0.880 (3)
C3	1.1814 (3)	0.5179 (2)	0.29853 (17)	0.0221 (5)	0.880 (3)
H3A	1.1627	0.5635	0.234	0.033*	0.880 (3)
H3B	1.2405	0.4534	0.2867	0.033*	0.880 (3)
H3C	1.237	0.5818	0.3547	0.033*	0.880 (3)
C4	1.0608 (2)	0.37797 (18)	0.42997 (13)	0.0276 (4)	
H4A	0.9649	0.3331	0.4503	0.041*	0.880 (3)
H4B	1.1163	0.4422	0.486	0.041*	0.880 (3)
H4C	1.1199	0.3134	0.4183	0.041*	0.880 (3)
C5	0.90750 (18)	0.43599 (16)	0.14037 (13)	0.0222 (3)	
H5A	0.8456	0.3773	0.0829	0.033*	0.880 (3)
H5B	1.0044	0.4764	0.1193	0.033*	0.880 (3)
H5C	0.8562	0.5047	0.1568	0.033*	0.880 (3)
C1'	0.9525 (13)	0.3419 (12)	0.3000 (11)	0.0135 (3)	0.120 (3)
H1'	1.0041	0.2825	0.2648	0.016*	0.120 (3)
C2'	0.9878 (13)	0.4706 (12)	0.2442 (10)	0.0161 (3)	0.120 (3)
H2'	0.9385	0.5336	0.2773	0.019*	0.120 (3)
C3'	1.164 (2)	0.530 (2)	0.2663 (15)	0.0221 (5)	0.120 (3)
H3'1	1.1961	0.5462	0.342	0.033*	0.120 (3)
H3'2	1.1869	0.6134	0.2315	0.033*	0.120 (3)
H3'3	1.2161	0.4683	0.239	0.033*	0.120 (3)
H4'1	1.044	0.2992	0.4706	0.041*	0.120 (3)
H4'2	1.0292	0.4487	0.4653	0.041*	0.120 (3)
H4'3	1.1677	0.4056	0.4242	0.041*	0.120 (3)
H5'1	0.9224	0.5132	0.0978	0.033*	0.120 (3)
H5'2	0.8003	0.4047	0.1438	0.033*	0.120 (3)
H5'3	0.945	0.3663	0.1085	0.033*	0.120 (3)
C11	0.72299 (16)	0.13544 (14)	0.07574 (11)	0.0160 (3)	
C12	0.84843 (17)	0.11312 (15)	0.03598 (12)	0.0193 (3)	
H12A	0.9257	0.0869	0.0815	0.023*	
C13	0.86207 (19)	0.12861 (16)	−0.06911 (13)	0.0229 (3)	
H13	0.9481	0.1128	−0.095	0.027*	
C14	0.7501 (2)	0.16710 (17)	−0.13625 (13)	0.0259 (3)	
H14	0.7596	0.1784	−0.2081	0.031*	
C15	0.6245 (2)	0.18891 (19)	−0.09798 (13)	0.0273 (4)	
H15A	0.5479	0.2155	−0.1438	0.033*	
C16	0.60969 (18)	0.17224 (17)	0.00689 (13)	0.0221 (3)	
H16	0.5222	0.1859	0.032	0.027*	
C21	0.82593 (16)	0.01717 (14)	0.25649 (12)	0.0160 (3)	
C22	0.78897 (18)	−0.10914 (15)	0.20857 (14)	0.0219 (3)	
H22	0.7106	−0.1304	0.1506	0.026*	
C23	0.8651 (2)	−0.20396 (16)	0.24445 (15)	0.0280 (4)	
H23A	0.8368	−0.29	0.2121	0.034*	
C24	0.9823 (2)	−0.17373 (18)	0.32730 (14)	0.0301 (4)	
H24A	1.0354	−0.2382	0.3515	0.036*	
C25	1.0208 (2)	−0.0491 (2)	0.37406 (14)	0.0335 (4)	
H25A	1.1018	−0.0272	0.4303	0.04*	
C26	0.9421 (2)	0.04519 (17)	0.33964 (13)	0.0251 (3)	
H26	0.9687	0.1301	0.3739	0.03*	
C31	0.50526 (16)	0.34906 (15)	0.22286 (11)	0.0160 (3)	
C32	0.49159 (18)	0.44964 (17)	0.15521 (13)	0.0223 (3)	
H32	0.5688	0.5275	0.1613	0.027*	
C33	0.3657 (2)	0.4373 (2)	0.07870 (13)	0.0296 (4)	
H33	0.3575	0.5064	0.033	0.035*	
C34	0.2534 (2)	0.3248 (2)	0.06951 (14)	0.0312 (4)	
H34	0.1684	0.3157	0.0167	0.037*	
C35	0.26411 (18)	0.22487 (18)	0.13728 (15)	0.0283 (4)	
H35	0.186	0.1477	0.1311	0.034*	
C36	0.38839 (17)	0.23698 (16)	0.21414 (13)	0.0208 (3)	
H36	0.3941	0.1687	0.2611	0.025*	
C41	0.60386 (16)	0.28426 (15)	0.42658 (12)	0.0172 (3)	
C42	0.48788 (18)	0.32576 (18)	0.46699 (13)	0.0245 (3)	
H42	0.4469	0.3932	0.4343	0.029*	
C43	0.43217 (19)	0.2696 (2)	0.55412 (14)	0.0293 (4)	
H43	0.3531	0.2983	0.5804	0.035*	
C44	0.49202 (19)	0.17178 (18)	0.60269 (13)	0.0272 (4)	
H44	0.4542	0.1332	0.6623	0.033*	
C45	0.60676 (19)	0.13068 (17)	0.56409 (13)	0.0245 (3)	
H45	0.6477	0.0636	0.5975	0.029*	
C46	0.66313 (17)	0.18625 (15)	0.47688 (12)	0.0195 (3)	
H46	0.7426	0.1572	0.4514	0.023*	

### Atomic displacement parameters (Å^2^)

**Table:**

	*U*^11^	*U*^22^	*U*^33^	*U*^12^	*U*^13^	*U*^23^
P1	0.01201 (17)	0.01326 (17)	0.01526 (18)	0.00541 (13)	0.00153 (13)	0.00045 (13)
P2	0.01198 (17)	0.01493 (18)	0.01806 (19)	0.00708 (13)	0.00105 (13)	−0.00021 (14)
N1	0.0110 (5)	0.0127 (5)	0.0190 (6)	0.0053 (4)	0.0011 (4)	−0.0007 (4)
C1	0.0109 (7)	0.0155 (7)	0.0148 (8)	0.0050 (5)	0.0015 (6)	−0.0005 (6)
C2	0.0141 (7)	0.0184 (8)	0.0165 (7)	0.0057 (6)	0.0015 (6)	−0.0021 (6)
C3	0.0164 (9)	0.0249 (10)	0.0232 (12)	0.0010 (7)	0.0034 (9)	−0.0015 (9)
C4	0.0360 (9)	0.0294 (9)	0.0188 (8)	0.0104 (7)	0.0042 (7)	0.0024 (7)
C5	0.0250 (8)	0.0243 (8)	0.0195 (7)	0.0126 (6)	−0.0003 (6)	−0.0008 (6)
N1'	0.0110 (5)	0.0127 (5)	0.0190 (6)	0.0053 (4)	0.0011 (4)	−0.0007 (4)
C1'	0.0109 (7)	0.0155 (7)	0.0148 (8)	0.0050 (5)	0.0015 (6)	−0.0005 (6)
C2'	0.0141 (7)	0.0184 (8)	0.0165 (7)	0.0057 (6)	0.0015 (6)	−0.0021 (6)
C3'	0.0164 (9)	0.0249 (10)	0.0232 (12)	0.0010 (7)	0.0034 (9)	−0.0015 (9)
C4'	0.0360 (9)	0.0294 (9)	0.0188 (8)	0.0104 (7)	0.0042 (7)	0.0024 (7)
C5'	0.0250 (8)	0.0243 (8)	0.0195 (7)	0.0126 (6)	−0.0003 (6)	−0.0008 (6)
C11	0.0162 (6)	0.0164 (7)	0.0159 (7)	0.0055 (5)	0.0015 (5)	−0.0002 (5)
C12	0.0185 (7)	0.0209 (7)	0.0211 (7)	0.0091 (6)	0.0041 (6)	0.0022 (6)
C13	0.0257 (8)	0.0245 (8)	0.0226 (8)	0.0104 (6)	0.0095 (6)	0.0020 (6)
C14	0.0325 (9)	0.0303 (9)	0.0172 (7)	0.0104 (7)	0.0061 (6)	0.0011 (6)
C15	0.0271 (8)	0.0380 (10)	0.0187 (8)	0.0144 (7)	−0.0012 (6)	0.0025 (7)
C16	0.0191 (7)	0.0303 (8)	0.0194 (7)	0.0118 (6)	0.0016 (6)	0.0006 (6)
C21	0.0174 (7)	0.0163 (7)	0.0173 (7)	0.0083 (5)	0.0054 (5)	0.0031 (5)
C22	0.0210 (7)	0.0176 (7)	0.0292 (8)	0.0071 (6)	0.0057 (6)	0.0004 (6)
C23	0.0345 (9)	0.0163 (7)	0.0393 (10)	0.0120 (7)	0.0151 (8)	0.0033 (7)
C24	0.0442 (10)	0.0321 (9)	0.0264 (9)	0.0288 (8)	0.0142 (8)	0.0127 (7)
C25	0.0425 (10)	0.0436 (11)	0.0218 (8)	0.0308 (9)	−0.0036 (7)	0.0015 (8)
C26	0.0323 (9)	0.0265 (8)	0.0197 (8)	0.0184 (7)	−0.0038 (6)	−0.0028 (6)
C31	0.0135 (6)	0.0217 (7)	0.0159 (7)	0.0102 (5)	0.0027 (5)	0.0001 (5)
C32	0.0197 (7)	0.0300 (8)	0.0217 (8)	0.0126 (6)	0.0063 (6)	0.0076 (6)
C33	0.0283 (9)	0.0485 (11)	0.0194 (8)	0.0233 (8)	0.0044 (7)	0.0101 (7)
C34	0.0235 (8)	0.0522 (11)	0.0216 (8)	0.0226 (8)	−0.0056 (6)	−0.0080 (8)
C35	0.0164 (7)	0.0313 (9)	0.0365 (10)	0.0100 (6)	−0.0037 (7)	−0.0117 (7)
C36	0.0166 (7)	0.0211 (7)	0.0267 (8)	0.0099 (6)	0.0017 (6)	−0.0015 (6)
C41	0.0163 (7)	0.0205 (7)	0.0149 (7)	0.0062 (5)	−0.0004 (5)	−0.0009 (5)
C42	0.0221 (8)	0.0346 (9)	0.0207 (8)	0.0148 (7)	0.0033 (6)	0.0016 (7)
C43	0.0228 (8)	0.0457 (11)	0.0222 (8)	0.0122 (7)	0.0062 (6)	0.0001 (7)
C44	0.0273 (8)	0.0352 (9)	0.0162 (7)	0.0009 (7)	0.0038 (6)	0.0009 (7)
C45	0.0299 (8)	0.0243 (8)	0.0178 (7)	0.0059 (6)	−0.0010 (6)	0.0018 (6)
C46	0.0210 (7)	0.0208 (7)	0.0173 (7)	0.0077 (6)	0.0000 (6)	−0.0009 (6)

### Geometric parameters (Å, °)

**Table:**

P1—N1	1.7219 (14)	C15—C16	1.389 (2)
P1—C21	1.8314 (16)	C15—H15A	0.95
P1—C11	1.8406 (17)	C16—H16	0.95
P2—N1	1.7279 (13)	C21—C26	1.382 (2)
P2—C41	1.8267 (17)	C21—C22	1.398 (2)
P2—C31	1.8367 (16)	C22—C23	1.387 (2)
N1—C1	1.516 (2)	C22—H22	0.95
C1—C2	1.547 (2)	C23—C24	1.386 (3)
C1—C5	1.559 (2)	C23—H23A	0.95
C1—H1	1.00	C24—C25	1.376 (3)
C2—C4	1.512 (2)	C24—H24A	0.95
C2—C3	1.531 (3)	C25—C26	1.392 (2)
C2—H2	1.00	C25—H25A	0.95
C3—H3A	0.98	C26—H26	0.95
C3—H3B	0.98	C31—C32	1.394 (2)
C3—H3C	0.98	C31—C36	1.398 (2)
C4—H4A	0.98	C32—C33	1.395 (2)
C4—H4B	0.98	C32—H32	0.95
C4—H4C	0.98	C33—C34	1.376 (3)
C5—H5A	0.98	C33—H33	0.95
C5—H5B	0.98	C34—C35	1.385 (3)
C5—H5C	0.98	C34—H34	0.95
C1'—C2'	1.531 (18)	C35—C36	1.387 (2)
C1'—H1'	1.00	C35—H35	0.95
C2'—C3'	1.59 (2)	C36—H36	0.95
C2'—H2'	1.00	C41—C46	1.393 (2)
C3'—H3'1	0.98	C41—C42	1.402 (2)
C3'—H3'2	0.98	C42—C43	1.388 (2)
C3'—H3'3	0.98	C42—H42	0.95
C11—C12	1.395 (2)	C43—C44	1.386 (3)
C11—C16	1.400 (2)	C43—H43	0.95
C12—C13	1.388 (2)	C44—C45	1.379 (3)
C12—H12A	0.95	C44—H44	0.95
C13—C14	1.387 (2)	C45—C46	1.390 (2)
C13—H13	0.95	C45—H45	0.95
C14—C15	1.386 (2)	C46—H46	0.95
C14—H14	0.95		
			
N1—P1—C21	106.57 (7)	C14—C15—C16	120.55 (15)
N1—P1—C11	104.94 (7)	C14—C15—H15A	119.7
C21—P1—C11	99.56 (7)	C16—C15—H15A	119.7
N1—P2—C41	104.98 (7)	C15—C16—C11	120.32 (15)
N1—P2—C31	106.17 (7)	C15—C16—H16	119.8
C41—P2—C31	99.78 (7)	C11—C16—H16	119.8
C1—N1—P1	121.49 (10)	C26—C21—C22	117.95 (14)
C1—N1—P2	115.52 (10)	C26—C21—P1	124.64 (12)
P1—N1—P2	117.83 (7)	C22—C21—P1	116.91 (12)
N1—C1—C2	112.09 (13)	C23—C22—C21	120.96 (16)
N1—C1—C5	112.51 (12)	C23—C22—H22	119.5
C2—C1—C5	109.68 (13)	C21—C22—H22	119.5
N1—C1—H1	107.4	C24—C23—C22	120.29 (16)
C2—C1—H1	107.4	C24—C23—H23A	119.9
C5—C1—H1	107.4	C22—C23—H23A	119.9
C4—C2—C3	109.39 (15)	C25—C24—C23	119.14 (15)
C4—C2—C1	113.19 (14)	C25—C24—H24A	120.4
C3—C2—C1	111.16 (15)	C23—C24—H24A	120.4
C4—C2—H2	107.6	C24—C25—C26	120.57 (17)
C3—C2—H2	107.6	C24—C25—H25A	119.7
C1—C2—H2	107.6	C26—C25—H25A	119.7
C2—C3—H3A	109.5	C21—C26—C25	121.07 (16)
C2—C3—H3B	109.5	C21—C26—H26	119.5
H3A—C3—H3B	109.5	C25—C26—H26	119.5
C2—C3—H3C	109.5	C32—C31—C36	118.56 (14)
H3A—C3—H3C	109.5	C32—C31—P2	117.02 (12)
H3B—C3—H3C	109.5	C36—C31—P2	124.42 (12)
C2—C4—H4A	109.5	C31—C32—C33	120.72 (16)
C2—C4—H4B	109.5	C31—C32—H32	119.6
H4A—C4—H4B	109.5	C33—C32—H32	119.6
C2—C4—H4C	109.5	C34—C33—C32	119.92 (17)
H4A—C4—H4C	109.5	C34—C33—H33	120
H4B—C4—H4C	109.5	C32—C33—H33	120
C1—C5—H5A	109.5	C33—C34—C35	120.06 (16)
C1—C5—H5B	109.5	C33—C34—H34	120
H5A—C5—H5B	109.5	C35—C34—H34	120
C1—C5—H5C	109.5	C34—C35—C36	120.32 (17)
H5A—C5—H5C	109.5	C34—C35—H35	119.8
H5B—C5—H5C	109.5	C36—C35—H35	119.8
C2'—C1'—H1'	104.3	C35—C36—C31	120.38 (16)
C1'—C2'—C3'	109.0 (11)	C35—C36—H36	119.8
C1'—C2'—H2'	107.2	C31—C36—H36	119.8
C3'—C2'—H2'	107.2	C46—C41—C42	118.29 (15)
C2'—C3'—H3'1	109.5	C46—C41—P2	124.54 (12)
C2'—C3'—H3'2	109.5	C42—C41—P2	116.92 (12)
H3'1—C3'—H3'2	109.5	C43—C42—C41	120.92 (16)
C2'—C3'—H3'3	109.5	C43—C42—H42	119.5
H3'1—C3'—H3'3	109.5	C41—C42—H42	119.5
H3'2—C3'—H3'3	109.5	C44—C43—C42	119.92 (16)
C12—C11—C16	118.48 (14)	C44—C43—H43	120
C12—C11—P1	123.86 (11)	C42—C43—H43	120
C16—C11—P1	117.61 (11)	C45—C44—C43	119.71 (16)
C13—C12—C11	121.00 (14)	C45—C44—H44	120.1
C13—C12—H12A	119.5	C43—C44—H44	120.1
C11—C12—H12A	119.5	C44—C45—C46	120.75 (16)
C14—C13—C12	120.00 (15)	C44—C45—H45	119.6
C14—C13—H13	120	C46—C45—H45	119.6
C12—C13—H13	120	C45—C46—C41	120.40 (15)
C15—C14—C13	119.63 (15)	C45—C46—H46	119.8
C15—C14—H14	120.2	C41—C46—H46	119.8
C13—C14—H14	120.2		
